# Mass Spectrometric Assays Reveal Discrepancies in Inhibition Profiles for the SARS‐CoV‐2 Papain‐Like Protease

**DOI:** 10.1002/cmdc.202200016

**Published:** 2022-02-17

**Authors:** Lennart Brewitz, Jos J. A. G. Kamps, Petra Lukacik, Claire Strain‐Damerell, Yilin Zhao, Anthony Tumber, Tika R. Malla, Allen M. Orville, Martin A. Walsh, Christopher J. Schofield

**Affiliations:** ^1^ Chemistry Research Laboratory, Department of Chemistry and the Ineos Oxford Institute for Antimicrobial Research University of Oxford 12 Mansfield Road OX1 3TA Oxford UK; ^2^ Diamond Light Source Ltd. Harwell Science and Innovation Campus OX11 0DE Didcot UK; ^3^ Research Complex at Harwell Harwell Science and Innovation Campus OX11 0FA Didcot UK

**Keywords:** Nucleophilic cysteine protease, PF-07321332/nirmatrelvir, SARS-CoV-2 papain-like protease/PL^pro^, SARS-CoV-2 main protease/M^pro^, viral protease inhibition

## Abstract

The two SARS‐CoV‐2 proteases, *i. e*. the main protease (M^pro^) and the papain‐like protease (PL^pro^), which hydrolyze the viral polypeptide chain giving functional non‐structural proteins, are essential for viral replication and are medicinal chemistry targets. We report a high‐throughput mass spectrometry (MS)‐based assay which directly monitors PL^pro^ catalysis *in vitro*. The assay was applied to investigate the effect of reported small‐molecule PL^pro^ inhibitors and selected M^pro^ inhibitors on PL^pro^ catalysis. The results reveal that some, but not all, PL^pro^ inhibitor potencies differ substantially from those obtained using fluorescence‐based assays. Some substrate‐competing M^pro^ inhibitors, notably PF‐07321332 (nirmatrelvir) which is in clinical development, do not inhibit PL^pro^. Less selective M^pro^ inhibitors, *e. g*. auranofin, inhibit PL^pro^, highlighting the potential for dual PL^pro^/M^pro^ inhibition. MS‐based PL^pro^ assays, which are orthogonal to widely employed fluorescence‐based assays, are of utility in validating inhibitor potencies, especially for inhibitors operating by non‐covalent mechanisms.

## Graphical abstract

**Double check**: A mass spectrometry‐based direct SARS‐CoV‐2 PL^pro^ assay reveals discrepancies in inhibition profiles for non‐covalent inhibitors. Use of assays orthogonal to the established fluorescence‐based assays is important for development of efficient small‐molecule PL^pro^ inhibitors.

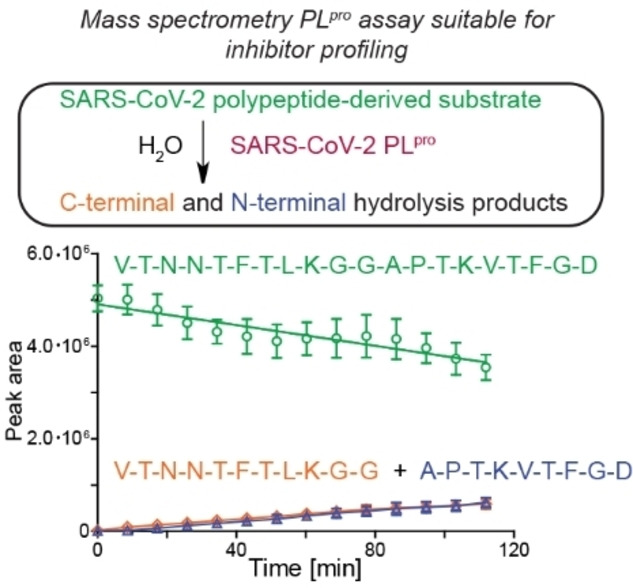

## Introduction

Despite the success of vaccination programs, severe acute respiratory syndrome coronavirus‐2 (SARS‐CoV‐2) remains a threat to human health at the beginning of 2022. Efficient and safe small‐molecule therapeutics approved to treat or prevent human SARS‐CoV‐2 infections, that complement vaccination efforts, are currently unavailable to the wider public. The two viral nucleophilic cysteine proteases, *i. e*. the papain‐like protease (PL^pro^; protease domain of the non‐structural protein 3, nsp3) and the main protease (M^pro^, nsp5), process the initially translated SARS‐CoV‐2 polypeptide chain into functional non‐structural proteins, which is essential for the viral life cycle.^[1]^ Therefore, PL^pro^ and M^pro^ are attractive targets to develop small‐molecule therapeutics.^[2]^ Targeting viral proteases has led to successful treatments for viral infections (*e. g*. by human immunodeficiency virus and hepatitis C virus),^[3]^ however, it is presently unclear whether PL^pro^ and/or M^pro^ inhibition (and/or inhibition of relevant human host proteases) is preferred for coronavirus disease 2019 (COVID‐19) prevention and/or treatment.

**Figure 1:**
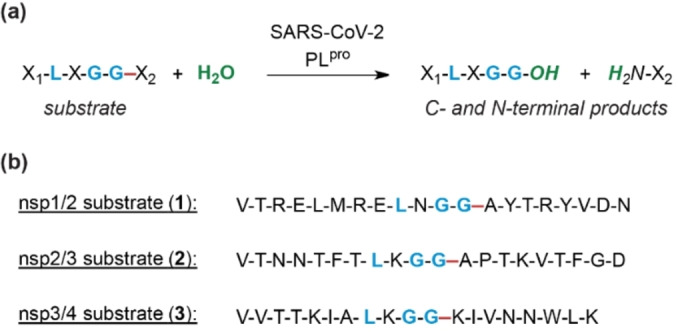
PL^pro^ catalysis and peptides used in this study. (**a**) Scheme for PL^pro^‐catalyzed hydrolysis of the SARS‐CoV‐2 polyprotein; (**b**) sequences of the SARS‐CoV‐2 polyprotein‐derived peptides used in this study, *i. e*. the nsp1/2 substrate (**1**), the nsp2/3 substrate (**2**), and the nsp3/4 substrate (**3**). Consensus sequence residues for PL^pro^ catalysis are in blue, the PL^pro^ cleavage site is in red.

Nsp3 is the largest SARS‐CoV‐2 nsp and contains, apart from its catalytically active PL^pro^ domain, other domains, including two ubiquitin (Ub)‐like domains, a SARS‐unique domain, and a nucleic acid binding domain, some of which are essential for catalytic activity.^[4]^ PL^pro^ catalyzes the hydrolytic cleavage of the SARS‐CoV‐2 polypeptide chain C‐terminal to LXGG motifs (Figure 1a).^[4b]^ Apart from releasing SARS‐CoV‐2 nsp1‐3, PL^pro^ is reported to modulate the host innate immune system by cleaving Ub and Ub‐like proteins (*e. g*. interferon stimulated gene 15, ISG15) from post‐translationally modified human host proteins, *i. e*. it has deubiquitinase type activity.^[5]^ By contrast, there is less evidence that M^pro^ accepts human substrates in a functionally relevant manner.^[6]^

Academic and industrial drug discovery campaigns have focused on developing small‐molecule SARS‐CoV‐2 M^pro^ inhibitors; considerably fewer studies have addressed PL^pro^ inhibition.[^2a^, ^2b^, ^2c^, ^2d^, ^2e^] At least to some extent, this might reflect limitations in current *in vitro* assays that monitor PL^pro^ catalysis. PL^pro^ assays have mostly employed fluorescence‐based methods that measure changes in absorbance/emission wavelengths upon cleavage of a C‐terminal fluorescent tag from a short substrate‐derived peptide, *e. g*. the cleavage of 7‐amino‐4‐methylcoumarin (Amc) from RLRGG‐Amc or related peptides, as originally reported for SARS‐CoV PL^pro^.^[7]^ Similar inhibition assays employing an entire protein domain attached to a fluorescent tag (*e. g*. ISG15‐Amc and Ub‐rhodamine) have also been reported,[^5a^, ^5b^] but are less commonly used, potentially due to their higher substrate costs. Förster (fluorescence) resonance energy transfer (FRET) assays have also been used to monitor PL^pro^ catalysis.^[8]^ Fluorescence‐based assays have benefits including, *e. g*., ease of operation, use of low protein/substrate concentrations, low substrate costs (at least in case of short fluorescence‐labelled peptides), and high sensitivity. However, common drawbacks of such assays include (i) the interference of UV‐active small‐molecules with fluorescence/emission signal(s) resulting in the identification of false positive hits, and (ii) the interaction of the artificial synthetic fluorescent tag with the protein, perturbing the substrate binding affinities.^[9]^

Application of PL^pro^ *in vitro* assays based on methods other than fluorescence is less frequent and those reported are mostly unsuitable for high‐throughput applications. They monitor PL^pro^ activity, *e. g*., by analyzing the cleavage of C‐ and N‐terminal protein‐tagged peptides using protein mass spectrometry (MS) and/or SDS‐PAGE.[^8a^, ^10^] A matrix‐assisted laser desorption/ionization time‐of‐flight (MALDI‐TOF) MS‐based deubiquitinase assay^[11]^ has been applied to investigate small‐molecules for PL^pro^ inhibition, however, this assay monitors only the deubiquitinase activity of PL^pro^ and requires additional sample manipulation for matrix formation.^[4a]^ Recently, we have reported a solid phase extraction (SPE) coupled to MS assay for SARS‐CoV‐2 M^pro^ which is suitable for screening small‐molecule inhibitors.^[12]^ We now report a high‐throughput SPE‐MS‐based SARS‐CoV‐2 PL^pro^ assay which uses oligopeptides as substrates and which is orthogonal to the typically employed fluorescence‐based PL^pro^ assays. The utility of this assay for the development of PL^pro^ inhibitors is shown by investigations on the effects of reported PL^pro^ inhibitors.

## Results and Discussion

### Development of a PL^pro^ Mass Spectrometry‐Based Assay

For developing a MS‐based assay to monitor PL^pro^ catalysis, a set of SPE‐MS compatible peptides, which mimic the reported SARS‐CoV‐2 PL^pro^ cleavage sites of nsp1/2 (VTRELMRELNGG/AYTRYVDN, **1**; ‘/’ indicates the PL^pro^ cleavage site), nsp2/3 (VTNNTFTLKGG/APTKVTFGD, **2**), and nsp3/4 (VVTTKIALKGG/KIVNNWLK, **3**), were designed based on the polyprotein sequence of the Wuhan‐Hu‐1 strain of SARS‐CoV‐2^[13]^ and synthesized with C‐terminal amides using solid phase peptide synthesis (SPPS) (Figure 1b, Supporting Figure S1).

The synthetic peptides were incubated in endpoint assays with PL^pro^ at 37 °C for 22 h; the reaction mixtures were then analyzed using SPE‐MS. The results reveal the nsp2/3 peptide (**2**) is a substantially more efficient PL^pro^ substrate compared to the nsp1/2 (**1**) and nsp3/4 (**3**) peptides, for which only low levels of hydrolyzed product peptides were observed (Supporting Figure S2). The reduced activity of PL^pro^ with **1** and **3** is consistent with prior reports that the isolated catalytic domain of SARS‐CoV‐2 PL^pro^ itself does not efficiently hydrolyze nsp1/2, but requires the presence of specific non‐catalytic domains present in nsp3,[^4a^, ^8a^, ^14^] and with similar observations for SARS‐CoV PL^pro^.^[15]^ The endpoint assays were used to identify preferred reaction conditions suitable for developing a direct SPE‐MS‐based PL^pro^ assay (0.2 μM PL^pro^ and 2.0 μM nsp2/3 peptide **2** in 50 mM Tris, pH 8.0).

**Figure 2:**
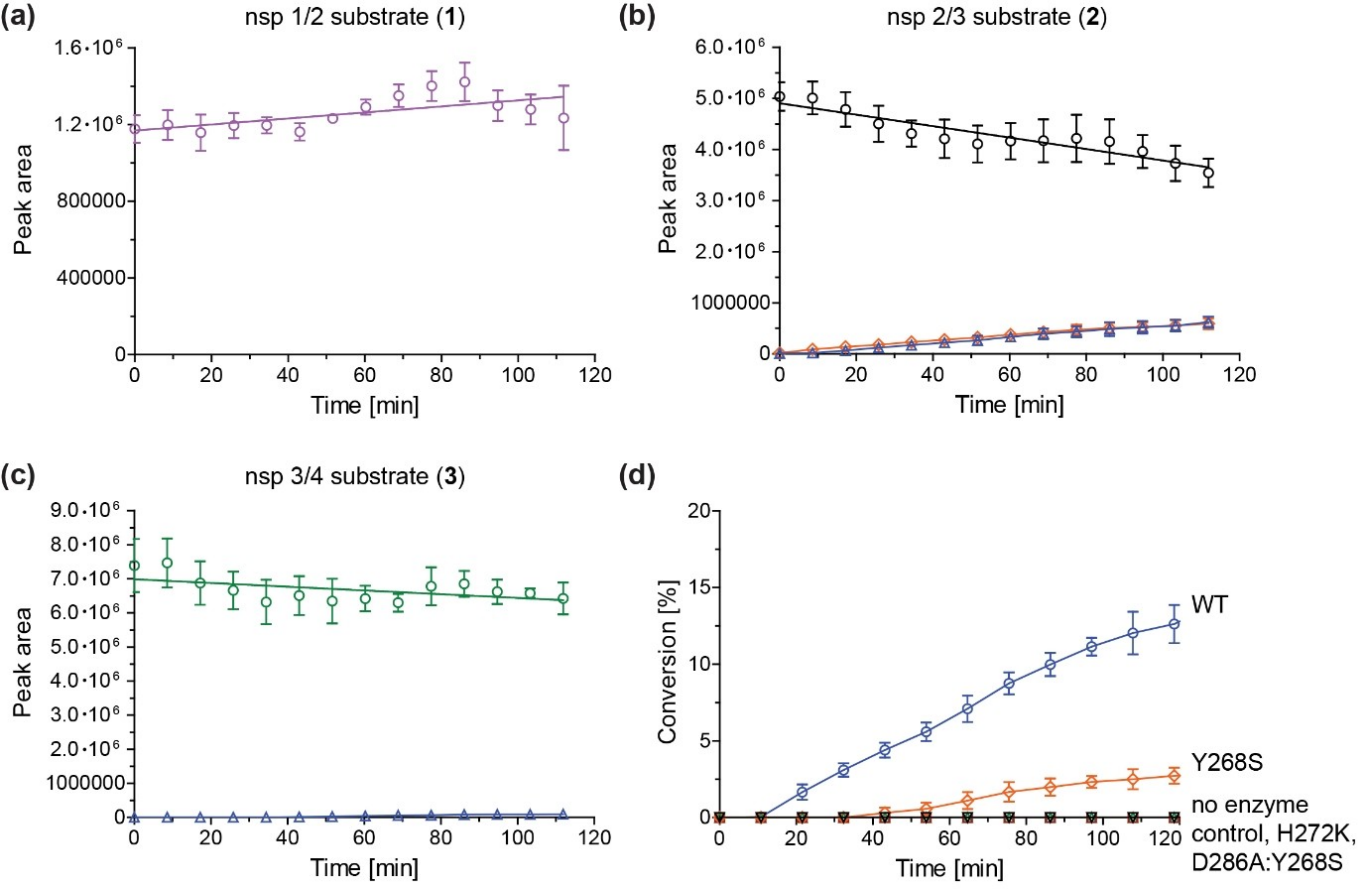
SPE‐MS can be employed to monitor PL^pro^ turnover *in vitro*. (**a**) Analysis of the peak areas of the extracted ion chromatograms for the SARS‐CoV‐2 nsp1/2 cleavage site‐derived peptide **1** (lavender circles) indicates no substantial PL^pro^‐catalyzed hydrolysis; the corresponding product peptides were not detected; (**b**) analysis of the peak areas of the extracted ion chromatograms for the SARS‐CoV‐2 nsp2/3 cleavage site‐derived peptide **2** (black circles) indicates PL^pro^‐catalyzed hydrolysis; levels of the C‐terminal (blue triangles) and N‐terminal (orange diamonds) product peptides increase; (**c**) analysis of the peak areas of the extracted ion chromatograms for the SARS‐CoV‐2 nsp3/4 cleavage site‐derived peptide **3** (green circles) indicates low levels of PL^pro^‐catalyzed hydrolysis; levels of the C‐terminal (blue triangles) product peptide increase; (**d**) monitoring hydrolysis of the nsp2/3 peptide **2** using SPE‐MS reveals that the H272K (green triangles) and D286A:Y268S (red squares) PL^pro^ active site variants exhibit no evidence for catalysis, whereas ∼15 % conversion was observed after 2 h for WT SARS‐CoV‐2 PL^pro^ (blue circles). The Y268S PL^pro^ variant (orange diamond) shows substantially reduced activity (<5 % conversion). Reactions were compared to a no enzyme control (black inverse triangles) and conversions were calculated based on the ratio of the N‐terminal hydrolysis product peptide and the N‐terminally acetylated N‐terminal product peptide, which was used as an internal standard. Results are means of three independent runs (n=3; mean ±standard deviation, SD).

Using the optimized reaction conditions, PL^pro^‐catalyzed substrate depletion/product formation was directly monitored using SPE‐MS at ambient temperature. In accord with the endpoint assays, turnover was clearly observed when using the nsp2/3 peptide (**2**) as substrate (Figure 2b), whereas only low levels of turnover were observed for the nsp3/4 (**3**) peptide (Figure 2c) and no hydrolysis of the nsp1/2 (**1**) peptide was detected (Figure 2a).

To quantify product formation, PL^pro^‐catalyzed reaction of nsp2/3 peptide **2** was performed in the presence of the corresponding N‐terminally acetylated N‐terminal and C‐terminal product peptides (0.2 μM each), which were used as internal standards. The presence of these peptides in the reaction mixture was shown not to interfere with PL^pro^ catalysis (Supporting Figure S3).

This assay set‐up was used to quantify the hydrolysis of nsp2/3 peptide **2**, catalyzed by wildtype (WT) PL^pro^ and its H272K, D286A:Y268S, and Y268S variants. Together with C111, PL^pro^ residues H272 and D286 form a catalytic triad; substitution of active site residues has been reported to ablate PL^pro^ catalytic activity.[^15b^, ^16^] The side chain of PL^pro^ Y268 is reported to interact with some substrate‐competing PL^pro^ inhibitors (*e. g*. GRL0617 and YM155);^[17]^ its direct interaction with substrates has not been observed crystallographically though it is proximate to the substrate binding site.[^5a^, ^5b^] The results reveal that the H272K and D286A:Y268S active site PL^pro^ variants are catalytically inactive (Figure 2d), confirming reported results.^[16]^ By contrast, the Y268S PL^pro^ variant showed catalytic activity, albeit at a reduced level compared to WT PL^pro^ (<5 % conversion after 2 h vs ∼15 % for WT; Figure 2d). It is probable that the substitution of the Y268 side chain for a serine side chain reduces productive PL^pro^ binding.

Next, the SPE‐MS assay was used to quantify the PL^pro^‐catalyzed hydrolysis of the nsp2/3 peptide (**2**) in the presence of either the nsp1/2 (**1**) peptide or the nsp3/4 (**3**) peptide in the same reaction vessel to investigate the effect of these two peptides on turnover. The results indicate that neither peptide **1** nor **3** affected the PL^pro^‐catalyzed hydrolysis of the nsp2/3 peptide (**2**) substantially (Supporting Figure S4).

The use of N‐terminally acetylated N‐terminal and C‐terminal product peptides as internal standards in the SPE‐MS assay enables, at least in principle, the determination of kinetic parameters for the PL^pro^‐catalyzed hydrolysis of peptides. However, it should be noted that high peptide concentrations are required to accurately determine kinetic parameters for substrates with a comparatively weak affinity for PL^pro^, which may result in the saturation of the ion detector and/or the suppression of product ionization, and thus perturb accurate measurements. The catalytic efficiency ($k_{\mathrm{cat}}$ /$K_{m}$ ) of PL^pro^ for the nsp2/3 peptide **2** was determined to be ∼64980 M^−1^ ⋅ s^−1^ using the SPE‐MS assay (Table 1, entry 1; Supporting Figure S5). Comparing the catalytic efficiency with reported $k_{\mathrm{cat}}$ /$K_{m}$ values obtained using fluorescence assays reveals that the PL^pro^ $k_{\mathrm{cat}}$ /$K_{m}$ value for **2** ranges between the $k_{\mathrm{cat}}$ /$K_{m}$ values reported when using ISG15‐Amc/K48‐linked Ub_2_‐Amc (521000 and 241000 M^−1^ ⋅ s^−1^, respectively;^[5a]^ Table 1, entries 2 and 3) and that when using RLRGG‐Amc (1840 M^−1^ ⋅ s^−1^;^[17b]^ Table 1, entry 4), possibly reflecting the higher biological relevance of the oligopeptide **2**. Note that the $k_{\mathrm{cat}}$ /$K_{m}$ value for **2** is similar to the $k_{\mathrm{cat}}$ /$K_{m}$ values obtained for ISG15‐TAMRA/K48‐linked Ub_2_‐TAMRA when using fluorescence polarization assays (Table 1, entries 5 and 6).^[5b]^

**Table 1:** Comparison of the SARS‐CoV‐2 PL^pro^ kinetic parameters obtained using SPE‐MS with those reported.

	Method	Substrate	$k_{\mathrm{cat}}$ /$K_{m}$ [M^−1^ ⋅ s^−1^]	$K_{m}$ [μM]
1	SPE‐MS^[a]^	nsp2/3 substrate **2**	64980 ±15256	49.4 ±10.5
2	Fluorescence^[5a]^	K48 linked Ub_2_‐Amc	241000±94000	61.23 ±19.76
3	Fluorescence^[5a]^	ISG15‐Amc	521000±36000	8.50 ±0.54
4	Fluorescence^[17b]^	RLRGG‐Amc	1840	not reported
5	Fluorescence^[14]^	Cbz‐RLRGG‐Amc	16000	6.9±1.4
6	Fluorescence^[18]^	Cbz‐RLRGG‐Amc	not reported	70.92 ±10.15
7	Fluorescence polarization^[5b]^	ISG15‐TAMRA	30210	not reported
8	Fluorescence polarization^[5b]^	K48 linked Ub_2_‐TAMRA	1634	not reported
9	FRET^[19]^	Dabcyl‐FTLKGGAPTKVTE‐Edans	1074.6±261.9	not reported
10	FRET^[8a]^	Abz‐FTLKGGAPTKVT−Y(3NO_2_)R	not reported	1854

[a] Kinetic parameters were determined as described in the Supporting Information (Section 4), Michaelis Menten kinetics are shown in Supporting Figure S5; results are means of three independent runs (n=3; mean ±SD). Abz: 2‐aminobenzoyl. Amc: 7‐amino‐4‐methylcoumarin. Cbz: −C(O)OCH_2_Ph. Dabcyl: 4‐([4‐(dimethylamino)phenyl]azo)benzoic acid. Edans: 5‐((2‐aminoethyl)amino)naphthalene‐1‐sulfonic acid. TAMRA: Carboxytetramethylrhodamine.

The Michaelis constant ($K_{m}$ value) for oligopeptide **2** is ∼6‐fold higher than that for ISG15‐Amc, the latter of which was determined using fluorescence‐based assays, but similar to that obtained for K48 linked Ub_2_‐Amc (Table 1, entries 1–3). The literature $K_{m}$ values for the RLRGG‐Amc peptide or related substrates from fluorescence‐based assays vary (Table 1, entries 4–6), but it appears that the $K_{m}$ value for **2** is lower than that for RLRGG‐Amc related substrates as supported by FRET assays with related substrates (Table 1, entries 9 and 10). This difference may reflect the ability of oligopeptide **2** to bind PL^pro^ remotely from the active site, including at the pockets C‐terminal of the cleavage site (*i. e*. S1′, S2′, etc.), potentially resulting in tighter binding than for the RLRGG‐Amc peptide. Binding of **2** is, however, substantially weaker than for substrates comprising a full protein domain, such as ISG15‐Amc.

### Mass Spectrometry‐Based PL^pro^ Inhibition Assays

PL^pro^ inhibition assays were performed in 384 well plates in the presence of the N‐terminally acetylated N‐terminal and C‐terminal product peptides to enable inhibition studies. The use of internal standards was found to be important to normalize conversion and, importantly, to account for inhibitor induced ion suppression of the product peptides (thus eliminating potential false‐positive hits) and well‐to‐well variations. Their use resulted in a robust assay, suitable for the determination of half‐maximum inhibitory concentrations (IC_50_‐values), manifesting generally high Z′‐factors (Supporting Figure S6). Inhibitors were used as DMSO solutions; the presence of DMSO (0.5 %_v/v_) in the inhibition assay did not substantially affect PL^pro^ activity.

**Figure 3:**
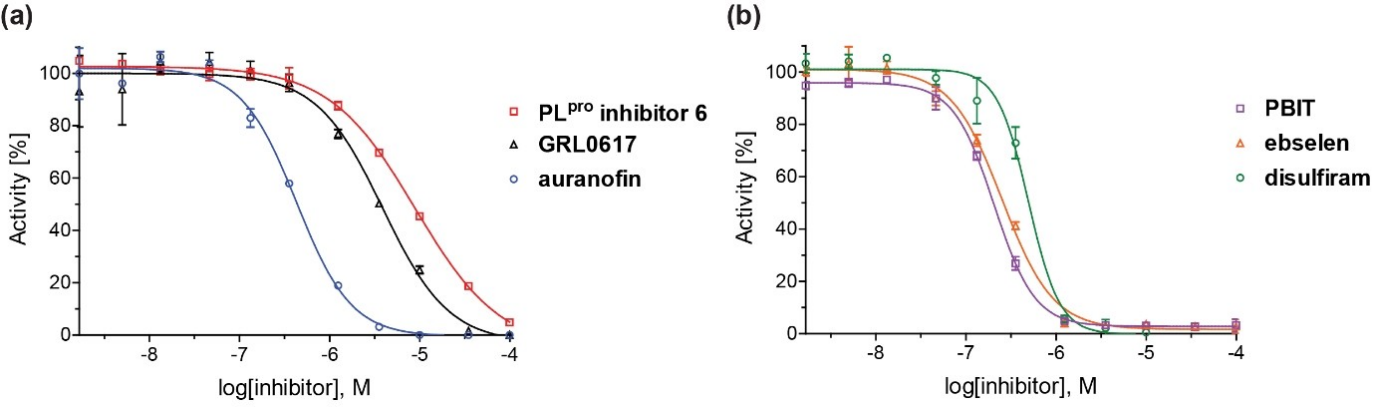
Representative dose‐response curves of selected PL^pro^ inhibitors. Representative dose‐response curves used to determine IC_50_‐values for the PL^pro^ inhibitors (**a**) GRL0617 (black triangles), PL^pro^ inhibitor 6 (red boxes), auranofin (blue circles), and (**b**) PBIT (lavender boxes), ebselen (orange triangles), disulfiram (green circles). Two dose‐response curves each composed of technical duplicates were independently determined using SPE‐MS PL^pro^ inhibition assays, performed as described in the Supporting Information (Section 5).

The SPE‐MS inhibition assay was then applied to investigate effects of selected reported PL^pro^ inhibitors on catalysis (Table 2). The results reveal that GRL0617, which was originally developed as a SARS‐CoV PL^pro^ inhibitor in 2008^[7a]^ and which was subsequently shown to efficiently inhibit SARS‐CoV‐2 PL^pro^ *in vitro* and in cells by non‐covalent binding at the P3 and P4 pockets as manifested by crystallographic studies,[^5a^, ^17b^] inhibits PL^pro^ in the SPE‐MS assay with similar potency to that reported using fluorescence‐based assays[^4a^, ^5d^, ^20^] (IC_50_∼3.8 μM, Table 2, entry 1). Analysis of the Hill coefficients of the inhibition curves reveals values in the range of −1, as predicted for single molecules competing with the substrate for binding (Figure 3a). Notably, a derivative of GRL0617, *i. e*. PL^pro^ inhibitor 6, is a less efficient inhibitor (IC_50_∼12.5 μM, Table 2, entry 2), in agreement with reported results obtained using fluorescence‐based assays,[^5d^, ^17a^, ^20a^] an observation which further validates the novel SPE‐MS assay.

**Table 2:** Use of the SPE‐MS PL^pro^ inhibition assay to investigate reported small‐molecule SARS‐CoV‐2 PL^pro^ inhibitors identified by fluorescence‐based assays.

	Reported PL^pro^ inhibitor	Reported IC_50_ [μM]^[a]^	SPE‐MS IC_50_ [μM]^[b]^		Reported PL^pro^ inhibitor	Reported IC_50_ [μM]^[a]^	SPE‐MS IC_50_ [μM]^[b]^
1	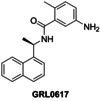	2.21^[4a]^ 2.4±0.2^[5d]^ 2.1±0.2^[20a]^ 1.61±0.09^[20b]^	3.8±1.0	8	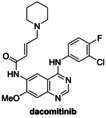	3–5^[8b]^	>50
2	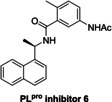	5.0±1.9^[5d]^ 11±3^[20a]^	12.5±6.1	9	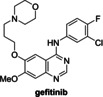	6–11^[8b]^	>50
3	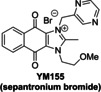	2.47±0.46^[17b]^	>50	10	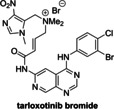	0.1–0.5^[8b]^	>50
4	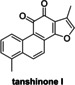	2.21±0.10^[17b]^	>50	11	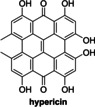	∼97 % inhibition at 100 μM^[25]^	>50
5	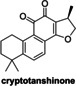	5.63±1.45^[17b]^	19.4±8.2	12	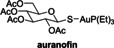	1.08^[4a]^ 0.75±0.13^[26]^	0.4±0.1
6	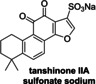	1.65±0.13^[22]^	>50	13	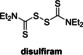	0.69^[4a]^ 1.05±0.34^[26]^	0.5±0.1
7	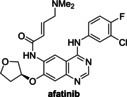	11–16^[8b]^	>50	14	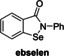	2.26±1.05^[27]^ 1.12±0.06^[28]^	0.3±0.1

[a] Reported IC_50_‐values were obtained using fluorescence‐based assays which monitor the PL^pro^‐catalyzed hydrolysis of Ac‐RLRGG‐Amc, RLRGG‐Amc, ISG15‐Amc, or related substrates, as described in the cited literature; [b] PL^pro^ SPE‐MS inhibition assays were performed using SPE‐MS as described in the Supporting Information (Section 5) employing 0.2 μM SARS‐CoV‐2 PL^pro^ and 2.0 μM of the nsp2/3 peptide **2** (VTNNTFTLKGG/APTKVTFGD) in the presence of N‐terminally acetylated product peptides (0.2 μM Ac‐VTNNTFTLKGG and 0.2 μM Ac‐APTKVTFGD). Inhibitors were commercially‐sourced and used as received. Results are means of two independent runs (n=2; mean±SD).

By contrast with the efficient PL^pro^ inhibition by GRL0617, the reported PL^pro^ inhibition by sepantronium bromide (YM‐155), a survivin suppressant compound under clinical investigation,^[21]^ was not observed using the SPE‐MS assay (Table 2, entry 3). A reported crystal structure of the C111S PL^pro^ active site variant in complex with YM‐155 indicates that the ligand binds non‐covalently near the P4 position in the substrate binding region.^[17b]^ Similarly to YM‐155, the natural product tanshinone I and its structural derivatives cryptotanshinone and tanshinone IIA sulfonate sodium, which are reported to efficiently inhibit PL^pro^ in fluorescence‐based assays,[^17b^, ^22^] did not, at least efficiently, inhibit in the SPE‐MS assays (Table 2, entries 4–6). Docking studies suggest, that tanshinone IIA sulfonate binds PL^pro^ non‐covalently near the active site and inhibits by substrate competition, which is in agreement with the reported small shift in the PL^pro^ melting temperature when incubated with the compound.^[22]^

The protein kinase inhibitors afatinib, dacomitinib, and gefitinib, which are approved human drugs for treatment of non‐small‐cell lung carcinoma,^[23]^ and the structurally‐related 4‐aminoquinazoline tarloxotinib bromide are reported PL^pro^ inhibitors, as identified in FRET‐based assays.^[8b]^ They were, however, all inactive in SPE‐MS assays (Table 2, entries 7–10). Note that pelitinib, another 4‐aminoquinazoline protein kinase inhibitor, which is structurally related to the afatinib drugs and which we did not test, is reported to be a potent allosteric inhibitor of the SARS‐CoV‐2 M^pro^, as shown by crystallographic analysis.^[24]^

The observed discrepancies in the inhibition results for some of the non‐covalently binding PL^pro^ inhibitors (YM‐155, tanshinone I and its derivatives, afatinib and related compounds) obtained using fluorescence‐based and SPE‐MS assays are likely a result of the different substrates used. Fluorescence‐based PL^pro^ assays have employed the relatively short RLRGG‐Amc peptide, or derivatives thereof, which, as supported by the reported kinetic parameters (Table 1), is not an efficient substrate and does not bind PL^pro^ as efficiently as its natural polypeptide substrates. In the SPE‐MS assays, a more biologically‐relevant 20mer oligopeptide was used as a substrate, which is, at least in principle, able to bind PL^pro^ more tightly as it also binds to the PL^pro^ substrate pockets C‐terminal of the cleavage site (*i. e*. S1′, S2′, etc), in agreement with the kinetic data (Table 1). The non‐covalent interactions of the inhibitors with PL^pro^ may be sufficiently strong to compete with the relatively short peptide substrates used in fluorescence‐based assays, but not be strong enough to efficiently compete with the tighter binding longer peptides used in the SPE‐MS assay.

Hypericin, which is reported to inhibit PL^pro^ catalysis in single concentration fluorescence‐based assays,^[25]^ also did not inhibit in SPE‐MS assays (Table 2, entry 11). The SPE‐MS assays, which directly monitor PL^pro^ catalysis, are thus of particular utility in validating the inhibition properties of molecules with an extended π‐electron system, such as hypericin, which can result in autofluorescence and/or fluorescence‐quenching and thus perturb the accuracy of fluorescence‐based assays.^[25]^

In summary, the SPE‐MS‐derived IC_50_‐values of reported small‐molecules PL^pro^ inhibitors, that inhibit via non‐covalent interactions, diverge in some (*e. g*. Table 2, entries 3–11), but not all (*e. g*. Table 2, entries 1 and 2), cases from those obtained using fluorescence‐based assays. By contrast, the SPE‐MS‐derived IC_50_‐values of some reported small‐molecule PL^pro^ inhibitors, that likely inhibit via covalent PL^pro^ modification, are generally in good agreement with those obtained using fluorescence‐based assays (Table 2, entries 12–14). Thus, the small‐molecules auranofin, which is an approved therapeutic for the treatment of rheumatoid arthritis,^[29]^ disulfiram, which is used to treat alcohol use disorder,^[30]^ and ebselen, all of which are reported to react covalently with proteins containing nucleophilic cysteines, including M^pro^,^[12]^ potently inhibit PL^pro^. It should, however, be noted that all of these compounds are likely not selective.

### Investigations on Potential Covalent PL^pro^ Inhibitors

Having validated the SPE‐MS SARS‐CoV‐2 PL^pro^ inhibition assay, it was applied to investigate selected small‐molecules reported to inhibit other cysteine proteases by covalent modification, including reported SARS‐CoV‐2 M^pro^ inhibitors (Table 3). The results reveal that 2‐(*para*‐tolyl)benzo[*d*]isothiazol‐3(2*H*)‐one (PBIT), which is a non‐specific covalent inhibitor of cysteine proteases including SARS‐CoV‐2 M^pro^,[^12^, ^31^] inhibits PL^pro^ efficiently (IC_50_∼0.3 μM; Table 3, entry 1 and Figure 3b), as anticipated based on the similar reactivity of PBIT, ebselen, and derivatives with M^pro^.[^28^, ^32^] By contrast, PF‐07321332 (nirmatrelvir), a selective SARS‐CoV‐2 M^pro^ inhibitor which is in clinical development and which inhibits covalently by reaction with the active site cysteine,^[2f]^ does not inhibit PL^pro^ (Table 3, entry 2). Similarly, MK‐0822 (odanacatib), a reported selective covalent inhibitor of the human cysteine protease cathepsin K,^[33]^ does not inhibit PL^pro^ (Table 3, entry 3), in accord with a recent report.^[34]^ SDZ‐224015, which is an investigational inhibitor of the human cysteine protease caspase‐1^[35]^ and which is also reported to efficiently inhibit SARS‐CoV‐2 M^pro^ by irreversible alkylation of the active site cysteine,^[8b]^ does not inhibit PL^pro^ (Table 3, entry 4), in agreement with its reported inability to inhibit PL^pro^.^[8b]^

**Table 3:** Investigating selected reported small‐molecule cysteine protease inhibitors for SARS‐CoV‐2 PL^pro^ inhibition using SPE‐MS inhibition assays.

	Cysteine protease inhibitor	IC_50_ [μM]^[a]^		Cysteine protease inhibitor	IC_50_ [μM]^[a]^
1	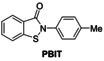	0.3±0.1	3	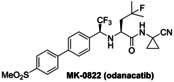	>50
2	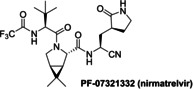	>20^[b]^	4	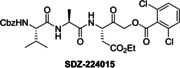	>50

[a] PL^pro^ SPE‐MS inhibition assays were performed using SPE‐MS as described in the Supporting Information (Section 5) employing 0.2 μM SARS‐CoV‐2 PL^pro^ and 2.0 μM of the nsp2/3 peptide **2** (VTNNTFTLKGG/APTKVTFGD) in the presence of N‐terminally acetylated product peptides (0.2 μM Ac‐VTNNTFTLKGG and 0.2 μM Ac‐APTKVTFGD). Inhibitors were commercially‐sourced and used as received, PF‐07321332^[2f]^ and SDZ‐224015^[8b]^ were synthesized as described. Results are means of two independent runs (n=2; mean±SD); [b] no PL^pro^ inhibition was observed at 20 μM PF‐07321332.

## Conclusion

Efficient small‐molecule PL^pro^ inhibitors are of potential use as COVID‐19 treatments and for functional assignment studies directed at improving current understanding of PL^pro^ biology, including its substrate preferences. Our MS‐based PL^pro^ assay is suitable for high‐throughput applications, including the screening of small‐molecules for PL^pro^ inhibition. The assay complements widely‐used fluorescence‐based PL^pro^ assays, to which it is orthogonal, and uses a simple oligopeptide as substrate that can be synthesized in a cost‐effective manner and that can be stored and handled without concerns regarding potential fluorescence quenching.

The SPE‐MS assay appears to be particularly useful for validation of small‐molecule PL^pro^ inhibitors identified using fluorescence‐based assays. In general, it appears that the SPE‐MS IC_50_‐values of covalently reacting PL^pro^ inhibitors, *e. g*. auranofin, disulfiram, and ebselen (Table 2, entries 12–14), correlate well with the reported values. By contrast, the SPE‐MS assay fails to confirm the potency of several, but not all, reported small‐molecule PL^pro^ inhibitors that work via non‐covalent modes. This might, to some extent, reflect the reduced binding affinity of the short peptide substrates (typically 5–12 mers) typically used in fluorescence‐based PL^pro^ assays. The longer 20mer oligopeptide used in our PL^pro^ assay is a more efficient substrate (Table 1) and is likely more biologically representative, in part because it also binds at the P′ sites. Consistent with this proposal, it is reported that residues flanking the substrate LXGG motif are important in enabling efficient SARS‐CoV PL^pro^ catalysis.^[16]^ Our results are in agreement with a recent study, which also raised concerns regarding the reported inhibition of PL^pro^ by certain non‐covalent inhibitors including YM‐155 and tanshinone I and its structural analogues.^[8c]^ Alternatively, the observed discrepancies in the IC_50_‐values of primarily non‐covalent inhibitors could be a result of false‐positive inhibitor identification in fluorescence‐based assays due to compound‐induced fluorescence quenching or compound autofluorescence, which are common drawbacks associated with such assays.^[9]^

Notably, the SPE‐MS assay was validated for identifying non‐covalent PL^pro^ inhibitors, such as GRL0617, which is possibly the best characterized PL^pro^ inhibitor, including by crystallography,[^5a^, ^17b^] and whose effect on PL^pro^ in cellular studies has been confirmed by multiple research groups.[^5a^, ^8a^, ^17^, ^20^] The structure of GRL0617 has been the basis for extensive structure‐activity relationship studies to develop a PL^pro^ inhibitor suitable for clinical use.[^17a^, ^20b^, ^36^] GRL0617 inhibits PL^pro^ with similar potency in both SPE‐MS and fluorescence‐based assays (Table 2, entry 1). The results highlight the importance of validating initial hits of PL^pro^ inhibitor screening campaigns using orthogonal assays and suggest that care should be taken when optimizing the potency of non‐covalent PL^pro^ inhibitors solely on the basis of fluorescence assays.

It should, however, be noted that a failure to observe PL^pro^ inhibition in the current SPE‐MS assay does not necessarily reflect the ability of small‐molecules to inhibit PL^pro^ activity, acting on different host or viral proteins and thus SARS‐CoV‐2 proliferation via different mechanisms.^[8c]^ The current SPE‐MS assay only efficiently detects inhibitors which bind to, or in the proximity of, the active site and thus compete with the substrate for PL^pro^ binding, or potentially those which bind allosterically and trigger conformational changes resulting in a loss of catalytic activity. Inhibition by other mechanisms, *e. g*. disrupting protein substrate binding distal from the active site, is not monitored by the current SPE‐MS assay. Hence, entire proteins or truncated domains thereof, such as, for example ISG15‐Amc, should be used as PL^pro^ substrates in assays to validate inhibition mechanisms and to identify allosteric types of inhibitors.

Different types of MS‐based assays have been employed to investigate the rate and stoichiometry of covalent SARS‐CoV‐2 M^pro^ modification by small‐molecules as well as the site of covalent M^pro^ modification;[^8b^, ^12^, ^32b^, ^37^] SPE‐MS PL^pro^ assays are likely suitable for similar investigations. The SPE‐MS assay for PL^pro^ described here complements that recently reported for M^pro^.^[12]^ It will thus help enable the development of inhibitors selective for PL^pro^ and/or M^pro^. The observation that compounds such as ebselen, disulfiram, and auranofin inhibit both PL^pro^ and M^pro^ highlights the possibility of dual action inhibitors (Table 2).^[28]^ Although these compounds may be insufficiently selective for widespread safe clinical use, at least disulfiram and auranofin are approved therapeutics for use in humans.[^29^, ^30^] We also found that potent substrate‐derived covalently reacting M^pro^ inhibitors, including PF‐07321332 (nirmatrelvir) which is in clinical use,[^2f^, ^38^] do not inhibit PL^pro^ (Table 3, entry 2), an observation of relevance to interpreting *in vivo/*clinical data.

## Conflict of interest

The authors declare no conflict of interest.

1

## Supporting information

As a service to our authors and readers, this journal provides supporting information supplied by the authors. Such materials are peer reviewed and may be re‐organized for online delivery, but are not copy‐edited or typeset. Technical support issues arising from supporting information (other than missing files) should be addressed to the authors.

Supporting Information

## Data Availability

The data that support the findings of this study are available in the supplementary material of this article.
