# Supplementary material for: Mass Spectrometric Assays Reveal Discrepancies in Inhibition Profiles for the SARS‐CoV‐2 Papain‐Like Protease
Source: ChemMedChem. 2022 Feb 17;17(9):e202200016. doi: 10.1002/cmdc.202200016 (PMC9015526; doi:10.1002/cmdc.202200016)
Supplement: Supplementary file 1 — Supporting Information [file CMDC-17-0-s001.pdf]

ChemMedChem

Supporting Information

Mass Spectrometric Assays Reveal Discrepancies in Inhibition Profiles for the SARS-CoV-2 Papain-Like Protease

Lennart Brewitz,* Jos J. A. G. Kamps, Petra Lukacik, Claire Strain-Damerell, Yilin Zhao, Anthony Tumber, Tika R. Malla, Allen M. Orville, Martin A. Walsh, and Christopher J. Schofield*

1. Supporting figures

Supporting Figure S1. The SARS-CoV-2 polypeptide chain-derived peptides used in this study (continues on the following 4 pages). The potential SARS-CoV-2 PL^{pro} substrate peptide sequences used based on the sequences of the reported PL^{pro} cleavage sites of the non-structural proteins 1, 2, and 3 (nsp1/2, nsp2/3, nsp3/4) of the SARS-CoV-2 Wuhan-Hu-1[1] strain. Peptides were synthesized with C-terminal amides by solid phase peptide synthesis (SPPS) and purified by HPLC as described in the Supporting Information (Section 3). The anticipated peptide masses and purity (>90%) were confirmed using solid phase extraction coupled to mass spectrometry (SPE-MS) in buffer (50 mM Tris, pH 8.0, 20 °C).

(**I**) (**a**) Sequence and purification characteristics of the SARS-CoV-2 nsp1/2 cleavage site-derived peptide **1** used in this work. The consensus sequence (LXGG) for PL^{pro} catalysis is in blue, the peptide cleavage site is in red; (**b**) mass spectrum (SPE-MS) of **1** (2.0 µM) in the reaction buffer (50 mM Tris, pH 8.0). m/z = 1178.57 corresponds to the +2 charge state of the nsp1/2 peptide **1**; the enlarged region shows the m/z +2 peak. m/z = 786.05 corresponds to the +3 charge state of **1**, m/z = 589.79 corresponds to the +4 charge state of **1**, and m/z = 122.08 corresponds to the +1 charge state of Tris. The peptide is estimated to be >90% pure based on HPLC analysis. Note, m/z values referred to are for the most abundant isotope.

(a)

SARS-CoV-2 nsp1/2 (**1**)

V-T-R-E-L-M-R-E-L-N-G-G–A-Y-T-R-Y-V-D-N

HPLC gradient: 0%$_{v/v}$ to 45%$_{v/v}$ MeCN/H_2O (+0.1%$_{v/v}$ TFA)
t_R: 21.6 min

SPE-MS: m/z calculated for $C_{100}H_{164}N_{32}O_{32}S$ $[M+2H]^{2+}$: 1178.5950, found: 1178.5669

(b)

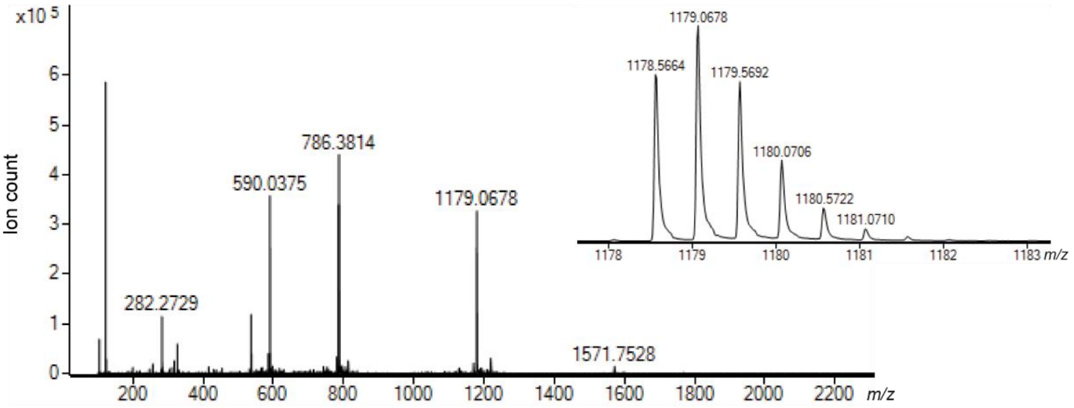

(**II**) (**a**) Sequence and purification characteristics of the SARS-CoV-2 nsp2/3 cleavage site-derived peptide **2** used in this work. The consensus sequence (LXGG) for PL^{pro} catalysis is in blue, the peptide cleavage site is in red; (**b**) mass spectrum (SPE-MS) of **2** (2.0 μM) in the reaction buffer (50 mM Tris, pH 8.0). m/z = 1034.03 corresponds to the +2 charge state of the nsp2/3 peptide **2**; the enlarged region shows the major m/z +2 peak. m/z = 2067.04 corresponds to the +1 charge state of **2**, m/z = 689.69 corresponds to the +3 charge state of **2**, and m/z = 122.08 corresponds to the +1 charge state of Tris. The peptide is estimated to be >90% pure based on HPLC analysis. Note, m/z values referred to are for the most abundant isotope.

(a)

SARS-CoV-2 nsp2/3 (**2**)

V-T-N-N-T-F-T- L-K-G-G–A-P-T-K-V-T-F-G-D

HPLC gradient: 0%$_{v/v}$ to 45%$_{v/v}$ MeCN/H_2O (+0.1%$_{v/v}$ TFA)
t_R: 20.2 min

SPE-MS: m/z calculated for $C_{92}H_{149}N_{25}O_{29}$ $[M+2H]^{2+}$: 1034.0471, found: 1034.0250

(b)

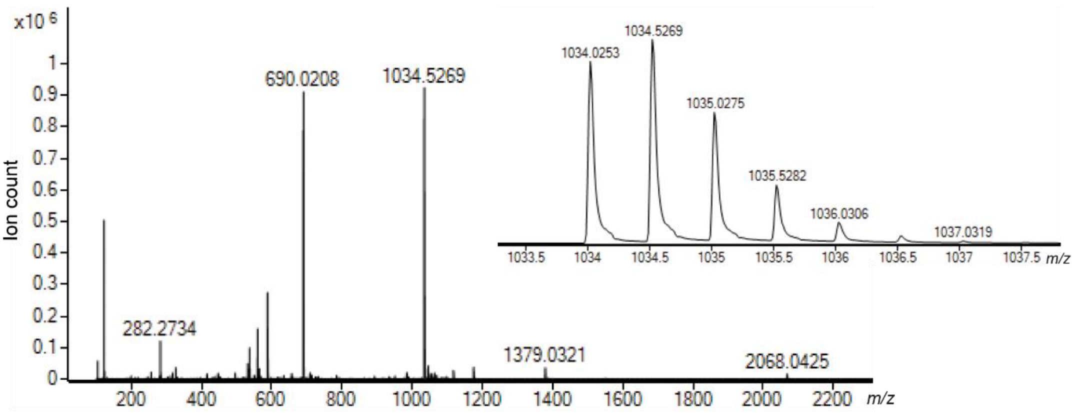

(**III**) (**a**) Sequence and purification characteristics of the SARS-CoV-2 nsp3/4 cleavage site-derived peptide **3** used in this work. The consensus sequence (LXGG) for PL^{pro} catalysis is in blue, the peptide cleavage site is in red; (**b**) mass spectrum (SPE-MS) of **3** (2.0 µM) in the reaction buffer (50 mM Tris, pH 8.0). The m/z = 1041.13 corresponds to the +2 charge state of the nsp3/4 peptide **3**; the enlarged region shows the m/z +2 peak. m/z = 694.42 corresponds to the +3 charge state of **3**, m/z = 521.09 corresponds to the +4 charge state of **3**, m/z = 417.06 corresponds to the +5 charge state of **3**, and m/z = 122.08 corresponds to the +1 charge state of Tris. The peptide is estimated to be >90% pure based on HPLC analysis. Note, m/z values referred to are for the most abundant isotope.

(a)

SARS-CoV-2 nsp3/4 (**3**)

V-V-T-T-K-I-A- L-K-G-G–K-I-V-N-N-W-L-K

HPLC gradient: 0%$_{v/v}$ to 45%$_{v/v}$ MeCN/H_2O (+0.1%$_{v/v}$ TFA)
t$_R$: 24.2 min

SPE-MS: m/z calculated for $C_{97}H_{171}N_{27}O_{23}$ $[M+2H]^{2+}$: 1041.1515, found: 1041.1280

(b)

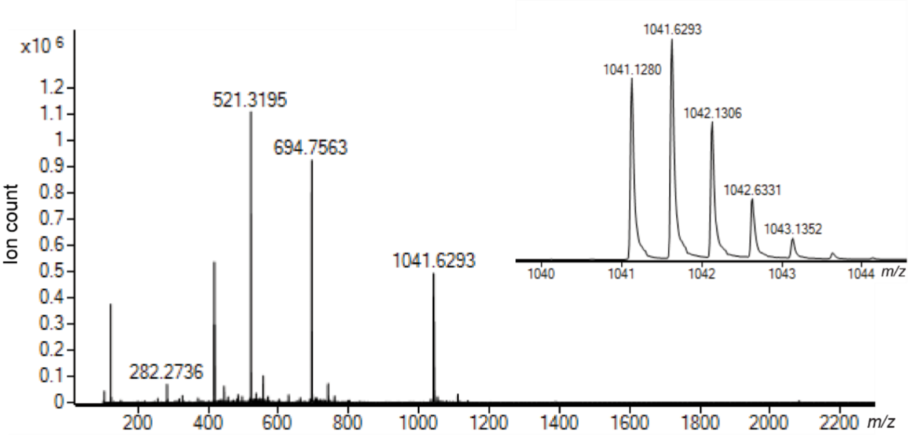

(**IV**) (**a**) Sequence and purification characteristics of the *N*-acetylated N-terminal hydrolysis product (Ac-VTNNTFTLKGG) of the PL^{pro}-catalyzed hydrolysis of the SARS-CoV-2 nsp2/3 cleavage site-derived peptide **2**; this peptide was used as an internal standard in this work; (**b**) mass spectrum (SPE-MS) of the *N*-acetylated N-terminal hydrolysis product of **2** (2.0 µM) in the reaction buffer (50 mM Tris, pH 8.0). m/z = 1192.62 corresponds to the +1 charge state of the *N*-acetylated N-terminal hydrolysis product; the enlarged region shows the major m/z +1 peak. m/z = 1214.61 corresponds to the +1 charge state of the peptide as sodium adduct, m/z = 596.82 corresponds to the +2 charge state of the peptide, and m/z = 122.08 corresponds to the +1 charge state of Tris. The peptide is estimated to be >90% pure based on HPLC analysis. Note, m/z values referred to are for the most abundant isotope.

(a)

N-Acetylated N-terminal hydrolysis product of nsp2/3 peptide **2**

Ac-V-T-N-N-T-F-T-L-K-G-G

HPLC gradient: 0%$_{v/v}$ to 45%$_{v/v}$ MeCN/H_2O (+0.1%$_{v/v}$ TFA) over 37 min

t$_R$: 20.0 min

SPE-MS: m/z calculated for $C_{52}H_{86}N_{15}O_{17}$ [M+H]$^+$: 1192.6321, found: 1192.6244

(b)

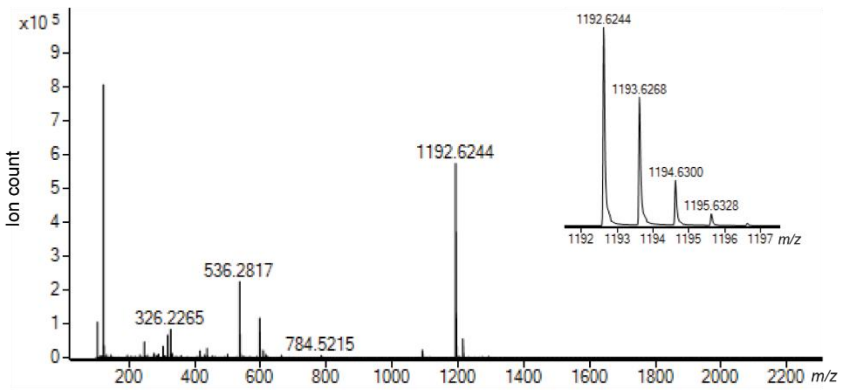

(**V**) (**a**) Sequence and purification characteristics of the *N*-acetylated C-terminal hydrolysis product of the PL^{pro}-catalyzed hydrolysis of the SARS-CoV-2 nsp2/3 cleavage site-derived peptide **2**; this peptide was used as an internal standard in this work; (**b**) mass spectrum (SPE-MS) of the *N*-acetylated C-terminal hydrolysis product of **2** (2.0 μM) in the reaction buffer (50 mM Tris, pH 8.0). The m/z = 976.50 corresponds to the +1 charge state of the *N*-acetylated C-terminal hydrolysis product; the enlarged region shows the major m/z +1 peak. m/z = 998.49 corresponds to the +1 charge state of the peptide as sodium adduct and m/z = 122.08 corresponds to the +1 charge state of Tris. The peptide is estimated to be >90% pure based on HPLC analysis. Note, m/z values referred to are for the most abundant isotope.

(a)

N-Acetylated C-terminal hydrolysis product of nsp2/3 peptide **2**

Ac-A-P-T-K-V-T-F-G-D

HPLC gradient: 0%$_{v/v}$ to 45%$_{v/v}$ MeCN/H_2O (+0.1%$_{v/v}$ TFA) over 37 min
t_R: 17.7 min
SPE-MS: m/z calculated for $C_{44}H_{70}N_{11}O_{14}$ $[M+H]^+$: 976.5098, found: 976.5040

(b)

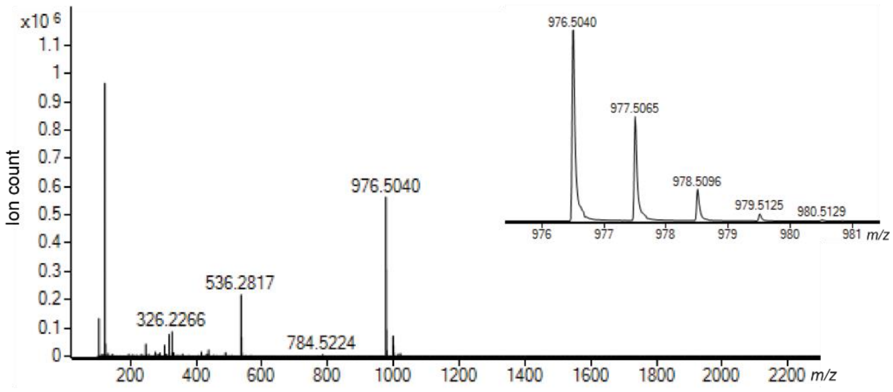

Supporting Figure S2. SARS-CoV-2 PL^{pro} endpoint assays reveal more efficient turnover of the SARS-CoV-2 nsp2/3 cleavage site-derived peptide than for the nsp1/2 or the nsp3/4 cleavage site-derived peptides. SARS-CoV-2 PL^{pro} assays monitoring the hydrolysis of (**a**) the SARS-CoV-2 nsp1/2 (VTRELMRELNGG/AYTRYVDN, **1**; Supporting Figure S1), (**b**) the SARS-CoV-2 nsp2/3 (VTNNTFTLKGG/APTKVTFGD, **2**; Supporting Figure S1), and (**c**) the SARS-CoV-2 nsp3/4 (VVTTKIALKGG/KIVNNWLK, **3**; Supporting Figure S1) cleavage site-derived peptides using SPE-MS reveal that the nsp2/3 peptide **2** is a more efficient substrate than the nsp1/2 **1** and nsp3/4 **3** peptides, in agreement with literature reports using related peptides.[2] Low levels of conversion were observed for the nsp3/4 peptide **3**, whereas no turnover was observed under the reaction conditions for the nsp1/2 peptide **1**. Conversion was observed to increase in a time-dependent manner, no peptide hydrolysis was detected in the absence of PL^{pro} after incubation for 22 h (*i.e.* no enzyme controls).

PL^{pro} activity assays (50 µL total reaction volume) were performed using 0.2 µM PL^{pro} and 2.0 µM substrate peptide in reaction buffer at 37 °C in 0.5 mL Eppendorf tubes with agitation (300 rpm). The enzyme reactions were stopped after the indicated time by addition of 10%$_{v/v}$ aqueous formic acid (5 µL). The reaction mixtures were transferred into a 384 well plate (Greiner) and PL^{pro}-catalyzed peptide cleavage was determined using SPE-MS as described in the Supporting Information (Section 4). An initial screen of buffer and reaction conditions revealed that PL^{pro} catalysis was most efficient in Tris buffer (50 mM Tris, pH = 8.0) in the absence of any additives such as NaCl. While PL^{pro} was active at ambient temperature (20 °C), conversion was higher at 37 °C. Note that conversions are estimates based on substrate depletion and thus not accurate, internal standards have not been used to quantify product formation.

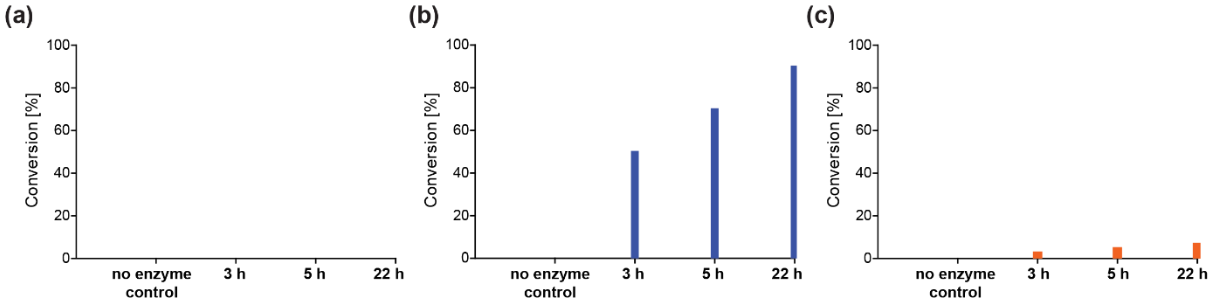

Supporting Figure S3. N-Terminal acetylated product peptides do not interfere with PL^{pro} catalysis and can be used as internal standards to quantify PL^{pro} turnover. The effect of the *N*-acetylated N-terminal and C-terminal product peptides of the PL^{pro}-catalyzed hydrolysis of the SARS-CoV-2 nsp2/3 cleavage site-derived peptide **2** (VTNNTFTLKGG/APTKVTFGD, Supporting Figure S1) on PL^{pro} catalysis was investigated using SPE-MS to investigate their utility as internal standards to quantify product formation. SPE-MS assays were performed in independent triplicates (n = 3; mean ± standard deviation, SD) as described in the Supporting Information (Section 4). Conditions: 0.2 μM PL^{pro}, 2.0 μM of the SARS-CoV-2 nsp2/3 cleavage site-derived peptide **2**, and 0.2 μM of the *N*-acetylated N-terminal product peptide (Ac-VTNNTFTLKGG; Supporting Figure S1) and/or the *N*-acetylated C-terminal product peptide (Ac-APTKVTFGD; Supporting Figure S1) in buffer (50 mM Tris, pH 8.0, 20 °C). Note that the Ac-APTKVTFGD peptide is not a PL^{pro} substrate; in the absence of the VTNNTFTLKGG/APTKVTFGD peptide in the reaction mixture, cleavage of the acetyl group from the Ac-APTKVTFGD peptide was not observed under the reaction conditions.

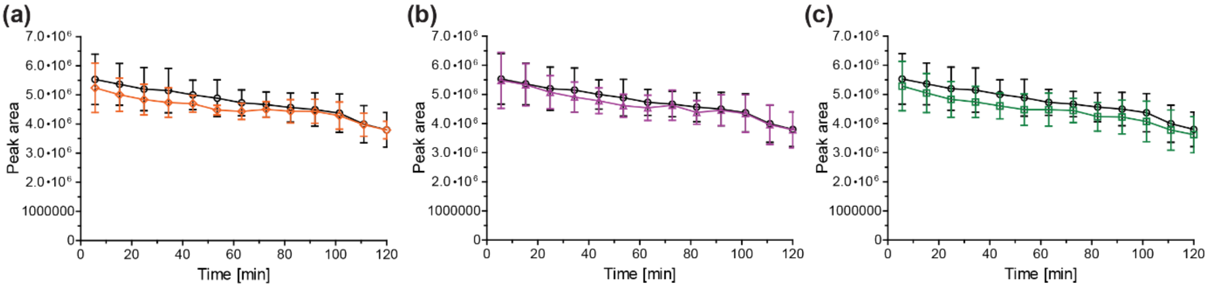

(**a**) Peak areas of the extracted ion chromatograms (m/z = +2) for the SARS-CoV-2 nsp2/3 cleavage site-derived peptide **2** in the absence (black circles) and the presence (orange diamonds) of the Ac-VTNNTFTLKGG peptide. The Ac-VTNNTFTLKGG peptide does not appear to affect PL^{pro} catalysis and may thus be used as internal standard; (**b**) peak areas of the extracted ion chromatograms (m/z = +2) for the SARS-CoV-2 nsp2/3 cleavage site-derived peptide **2** in the absence (black circles) and the presence (lavender triangles) of the Ac-APTKVTFGD peptide. The Ac-APTKVTFGD peptide does not appear to affect PL^{pro} catalysis and may thus be used as internal standard; (**c**) peak areas of the extracted ion chromatograms (m/z = +2) for the SARS-CoV-2 nsp2/3 cleavage site-derived peptide **2** in the absence (black circles) and the presence (green boxes) of both the Ac-VTNNTFTLKGG peptide and the Ac-APTKVTFGD peptide used as internal standards. The results reveal that PL^{pro}-catalyzed cleavage of the SARS-CoV-2 nsp2/3 cleavage site-derived peptide **2** can be performed in the presence of the Ac-VTNNTFTLKGG peptide and the Ac-APTKVTFGD peptide without altering the reaction profile, thus enabling quantification of product formation by comparison of the integrals of the product peptide ion counts with those of the *N*-acetylated product peptides present in the reaction mixture as inert internal standards.

Supporting Figure S4. The SARS-CoV-2 nsp1/2 and nsp3/4-derived peptides do not affect the PL^{pro}-catalyzed hydrolysis of the nsp2/3-derived peptide. The reaction profile of the PL^{pro}-catalyzed hydrolysis of the SARS-CoV-2 nsp2/3-derived peptide **2** does not alter substantially in the presence of equimolar amounts of either the nsp1/2-derived peptide **1** (blue boxes) or the nsp3/4-derived peptide **3** (orange triangles) in the same reaction vessel, as revealed by comparison with a control reaction containing neither **1** or **3** (black inverse triangles). Conversions of the SARS-CoV-2 nsp2/3-derived peptide **2** were determined by comparison of the peak areas of the extracted ion chromatograms (m/z = +2) for the N-terminal product peptide (VTNNTFTLKGG) with the one for the corresponding *N*-acetylated N-terminal product peptide, which was used as internal standard.
SPE-MS assays were performed in independent triplicates (n = 3; mean ± SD) as described in the Supporting Information (Section 4). Conditions: 0.2 μM PL^{pro}, 2.0 μM of the SARS-CoV-2 nsp2/3 cleavage site-derived peptide **2** (VTNNTFTLKGG/APTKVTFGD, Supporting Figure S1), 0.2 μM of the *N*-acetylated N-terminal product peptide (Ac-VTNNTFTLKGG; Supporting Figure S1) and the *N*-acetylated C-terminal product peptide (Ac-APTKVTFGD; Supporting Figure S1) in buffer (50 mM Tris, pH 8.0, 20 °C), and, if applicable, 2.0 μM of the SARS-CoV-2 nsp1/2 cleavage site-derived peptide **1** (VTRELMRELNGG/AYTRYVDN; Supporting Figure S1) or the SARS-CoV-2 nsp3/4 cleavage site-derived peptide **3** (VVTTKIALKGG/KIVNNWLK; Supporting Figure S1).

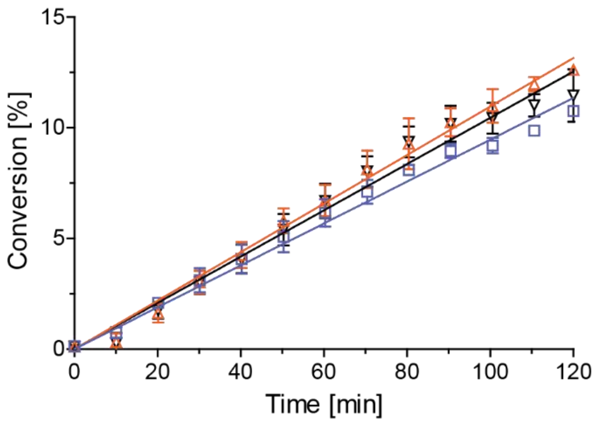

Supporting Figure S5. SARS-CoV-2 PL^{pro} substrate kinetic parameters for the SARS-CoV-2 nsp2/3 cleavage site-derived peptide. The maximum velocity (v_{max}) and Michaelis constant (K_m) of PL^{pro} were determined in independent triplicates (n = 3; mean ± SD) for the SARS-CoV-2 nsp2/3 cleavage site-derived peptide **2** (VTNNTFTLKGG/APTKVTFGD, Supporting Figure S1), monitoring PL^{pro}-catalyzed formation of the C-terminal APTKVTFGD product peptide by SPE-MS as described in the Supporting Information (Section 4). Conditions: 0.2 µM PL^{pro} and variable concentrations of the SARS-CoV-2 nsp2/3 cleavage site-derived peptide **2** (0.5 – 128 µM), as indicated, in buffer (50 mM Tris, pH 8.0, 20 °C). Reactions were performed in the presence of the *N*-acetylated N-terminal product peptide (Ac-VTNNTFTLKGG; Supporting Figure S1) and the *N*-acetylated C-terminal product peptide (Ac-APTKVTFGD; Supporting Figure S1), used as internal standards to quantify substrate turnover (0.2 µM of each *N*-acetylated peptide was used for substrate concentrations ranging from 0.5 – 2.0 µM, 1.0 µM of each *N*-acetylated peptide was used for substrate concentrations ranging from 4.0 – 8.0 µM, and 2.0 µM of each *N*-acetylated peptide was used for substrate concentrations ranging from 16 – 128 µM). Note that quantifying the turnover of the N-terminal VTNNTFTLKGG product peptide did not result in useful data, an observation which might relate to possible non-linear behavior of the VTNNTFTLKGG peptide at higher concentrations (the non-acetylated VTNNTFTLKGG peptide was not synthesized).

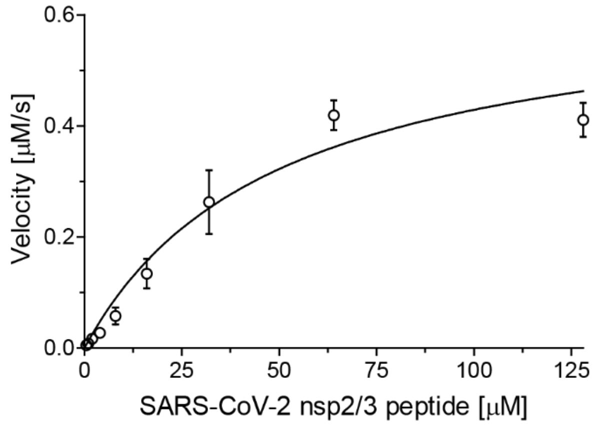

Michaelis Menten kinetics of PL^{pro} for the SARS-CoV-2 nsp2/3 cleavage site-derived peptide **2** afford a v_{max} of 0.642 ± 0.0637 $\mu M \cdot s^{-1}$ and a K_m of 49.4 ± 10.5 µM (goodness of fit: $R^2 = 0.9496$). Based on the assumption that the PL^{pro} used was completely active, the k_{cat} was calculated from the v_{max} to be 3.21 ± 0.32 s^{-1}. k_{cat} and K_m are in the range of those reported for ISG15-Amc and K48 linked Ub_2-Amc.[3]

The specificity constant (k_{cat}/K_m) was calculated to be 64980 ± 15256 $M^{-1} \cdot s^{-1}$, which is in the range of those reported using fluorescence-based PL^{pro} assays as shown in Table 1.

Supporting Figure S6. Robustness of the PL^{pro} SPE-MS inhibition assays. SPE-MS inhibition assays were performed as described in the Supporting Information (Section 5). Z'-factors[4] for inhibition assay plates analyzed to determine IC_{50}-values. The Z'-factors >0.5 (grey line) indicate a stable and robust assay of high quality.[4] Z'-factors were determined according to the literature using Microsoft Excel.[4]

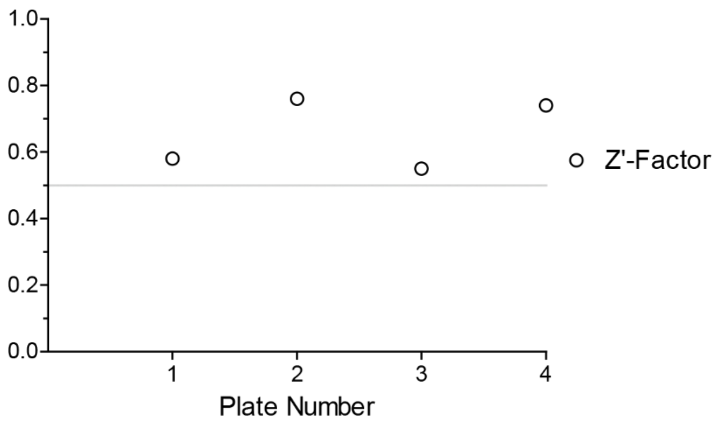

2. SARS-CoV-2 PL^{pro} production and purification

The PL^{pro} domain of nsp3 (region E746-T1063) was amplified from a synthetic fragment (Integrated DNA Technologies Ltd.) with In-Fusion adapted primers and cloned into a pOPINF derivative vector, containing an N-terminal hexa-histidine tag followed by a SUMO tag (ubiquitin like protein SMT3). The resultant plasmid was transformed into Lemo21(DE3) competent *E. coli* (NEB). Multiple transformant colonies were used to inoculate a starter culture supplemented with 0.5 mM rhamnose, 100 µg/mL carbenicillin and 34 µg/mL chloramphenicol. The culture was then grown to log phase (200 rpm, 6 h, 37 °C). 10 mL of the starter culture was used to inoculate 1 L of auto induction medium (Formedium) supplemented with 10 mL of glycerol and 100 µg/mL carbenicillin. The cultures were grown at 200 rpm, 37 °C, for 5 h then switched to 18 °C for 72 h. The cells were harvested by centrifugation and stored at -80 °C.

Approximately 40 g of cells were re-suspended in the purification buffer (50 mM Tris, pH 8, 300 mM NaCl) containing 10 mM imidazole and 0.03 µg/mL benzonase. Cells were lysed using an Avestin Emulsiflex homogeniser (3 passes, 30 kpsi, 4 °C). The lysate was centrifuged (50 000 g, 1 h, 4 °C). 10 mL of 50% His60 Ni Superflow Resin (Takara) was then added to the sample and stirred (1 h, 4 °C). The sample was applied to a gravity flow column, washed extensively using purification buffer containing 25 mM imidazole, and the protein was eluted using purification buffer containing 500 mM imidazole. Ubiquitin-like-specific protease 1 (Ulp1 protease) was added to the eluted protein at a ratio of 1:50 (w/w) to cleave the SUMO tag. The mixture was dialyzed against purification buffer containing 0.5 mM tris(2-carboxyethyl)phosphine (TCEP) (overnight, 4 °C). The protease and other impurities were removed from the cleaved target protein by reverse Nickel-NTA. PL^{pro} was further purified by gel filtration chromatography using a S75 16/600 pg (Cytiva) column equilibrated in purification buffer. Relevant fractions were pooled, concentrated, flash frozen in aliquots and stored at -80 °C. The enzyme was >95% pure as analyzed by SDS-PAGE and MS analysis and had the anticipated mass as reported; note that fresh aliquots, which were not frozen more than once, were used for inhibition assays.

The PL^{pro} variants, *i.e.* H272K (nsp3 position H1017), D286A:Y268S (nsp3 D1031 and Y1013), Y268S (nsp3 Y1013) PL^{pro}, were generated by overlap extension PCR and cloned by In-Fusion into the same pOPINF derivative vector as the wild type construct. The PL^{pro} variants were produced and purified in a similar manner as described above.

3. Peptide synthesis

The PL^{pro} substrate peptides mimicking the SARS-CoV-2 nsp1/2 (**1**), nsp2/3 (**2**), and nsp 3/4 (**3**) cleavage sites and the corresponding nsp2/3 (**2**)-derived *N*-acetylated C-terminal and N-terminal product peptides, which were used as internal standards, were prepared as C-terminal amides by solid phase peptide synthesis (SPPS) using commercially-sourced Fmoc-protected amino acids (Sigma-Aldrich, Inc.; Fluorochem Ltd.), peptide synthesis grade DMF (Sigma-Aldrich, Inc.), 20%$_{v/v}$ piperidine in DMF (AGTC Bioproducts Ltd.), HPLC-grade acetonitrile (Sigma-Aldrich, Inc), *N*,*N*-diisopropylcarbodiimide (Fluorochem Ltd.), oxyma (Sigma-Aldrich, Inc.) and Hünig's base (Sigma-Aldrich, Inc.). Microwave-assisted SPPS was performed using an automated peptide synthesizer (Liberty Blue, CEM Microwave Technology Ltd.) from the C- to N-terminus on Rink Amide MBHA resin (AGTC Bioproducts Ltd.; loading: 0.6-0.8 mmol/g) using iterative coupling (90 °C; 140 s; Fmoc-protected amino acids and *N*,*N*-diisopropylcarbodiimide, oxyma, Hünig's base) and deprotection steps (90 °C; 90 s; 20%$_{v/v}$ piperidine in DMF). For the synthesis of *N*-acetylated peptides to be used as internal standards, the free N-terminal amino group was capped after the final Fmoc-deprotection step using commercially-sourced *N*-acetoxysuccinimide (Tokyo Chemical Industry UK Ltd.) in DMF while the peptide was still immobilized on the resin.

After washing the resin-bound peptides with dichloromethane, they were cleaved from the resin and simultaneously deprotected using a mixture of trifluoroacetic acid, triisopropylsilane, 1,3-dimethoxybenzene, and

water (92.5/2.5/2.5/2.5%$_{v/v}$, respectively). Solids were separated; the remaining clear solution was diluted with diethyl ether (45 mL/0.1 mmol resin). After incubation for 30 min at 0 °C, the mixture was centrifuged for 10 min using a Beckman Coulter Allegra X-30R centrifuge equipped with a SX4400 rotor (4500 rpm) and the supernatant discarded. The solid residue was dissolved in a water/acetonitrile mixture, frozen using liquid N_2, and then lyophilized. The dried crude product was dissolved in a water/acetonitrile mixture, filtered, and purified using a semi-preparative HPLC machine (Shimadzu UK Ltd.) equipped with a reverse phase column (Gemini 00G-4454-U0-AX; phase: NX-C18). A linear gradient (typically 0−45%$_{v/v}$ over 35 min) of acetonitrile in MQ-grade water (each containing 0.1%$_{v/v}$ trifluoroacetic acid) was used as eluent. Fractions were analyzed by SPE-MS and those containing the pure peptide were combined and lyophilized. Sequences, mass spectra, and HPLC retention times for the peptides synthesized are shown in Supporting Figure S1.

4. PL^{pro} SPE-MS assays

SARS-CoV-2 PL^{pro} endpoint turnover assays were performed using 0.2 µM PL^{pro} and 2.0 µM substrate peptide in reaction buffer at 20 ºC or 37 ºC in 0.5 mL Eppendorf tubes with agitation (300 rpm). The enzyme reactions were stopped by addition of 10%$_{v/v}$ aqueous formic acid (5 µL). The reaction mixtures were transferred into a 384-well polypropylene assay plate (Greiner) and analyzed by SPE-MS.

SARS-CoV-2 PL^{pro} turnover assays for kinetic and competition experiments were performed in 96-well polypropylene assay plates (Greiner) with either a 1.0 or 0.5 mL final reaction volume; PL^{pro} catalysis was directly monitored using SPE-MS. The RapidFire RF 365 high-throughput sampling robot used was programmed to aspirate samples from the reaction mixture at the indicated time intervals.

MS-analyses were performed using a RapidFire RF 365 high-throughput sampling robot (Agilent) attached to an iFunnel Agilent 6550 accurate mass quadrupole time-of-flight (Q-TOF) mass spectrometer operated in the positive ionization mode. Assay samples were aspirated under vacuum for 0.6 s and loaded onto a C4 solid phase extraction (SPE) cartridge. After loading, the C4 SPE cartridge was washed with 0.1%$_{v/v}$ aqueous formic acid to remove non-volatile buffer salts (5.5 s, 1.5 mL/min). The peptide was eluted from the SPE cartridge with 0.1%$_{v/v}$ aqueous formic acid in 85/15$_{v/v}$ acetonitrile/water into the mass spectrometer (5.5 s, 1.25 mL/min) and the SPE cartridge re-equilibrated with 0.1%$_{v/v}$ aqueous formic acid (0.5 s, 1.25 mL/min). The mass spectrometer parameters were: capillary voltage (4000 V), nozzle voltage (1000 V), fragmentor voltage (365 V), gas temperature (280 °C), gas flow (13 L/min), sheath gas temperature (350 °C), sheath gas flow (12 L/min).

For data analysis, the m/z +2 charge states of the SARS-CoV-2 nsp1/2 (**1**), nsp2/3 (**2**), and nsp 3/4 (**3**) cleavage site-derived PL^{pro} substrate peptides and/or the m/z +1 charge states of the corresponding product peptides were used to extract ion chromatogram data; peak areas were integrated using the RapidFire Integrator software (Agilent). To quantify product formation, the m/z +1 charge states of both the C-terminal and the N-terminal product peptides of the PL^{pro}-catalyzed hydrolysis of the nsp2/3 peptide (**2**), as well as the corresponding *N*-acetylated C-terminal and N-terminal product peptides, which were used as internal standards, were used to extract ion chromatogram data; peak areas were integrated using the RapidFire Integrator software (Agilent). Data were exported into Microsoft Excel and used to calculate the product peptide concentrations using the equation: peptide concentration = 0.2 µM × (integral C- or N-terminal product peptide) / (integral *N*-acetylated C- or N-terminal product peptide).

5. PL^{pro} inhibition assays

SPE-MS PL^{pro} inhibition assays were performed using purified recombinant SARS-CoV-2 PL^{pro} and the SARS-CoV-2 nsp2/3 cleavage site-derived peptide **2** (VTNNTFTLKGG/APTKVTFGD, Supporting Figure S1). Note that fresh PL^{pro} aliquots, which were thawed and frozen not more than twice, were used for inhibition assays.

Solutions of the inhibitors (100% DMSO) were dry dispensed across 384-well polypropylene assay plates (Greiner) in an approximate three-fold and 11-point dilution series (100 µM top concentration) using an ECHO 550 acoustic dispenser (Labcyte). DMSO and formic acid were used as negative and positive inhibition controls, respectively. The final DMSO concentration was kept constant at $0.5\%_{v/v}$ throughout all experiments (using the DMSO backfill option of the acoustic dispenser). Each reaction was performed in technical duplicates in adjacent wells of the assay plates; additionally, assays were performed in independent duplicates (n = 2).

The Enzyme Mixture (25 µL/well), containing SARS-CoV-2 PL^{pro} (0.4 µM) in 50 mM Tris buffer (pH 8.0), was dispensed across the inhibitor-containing 384-well assay plates with a multidrop dispenser (ThermoFischer Scientific) at 20 °C under an ambient atmosphere. The plates were subsequently centrifuged (1000 rpm, 5 s) and incubated for 15 min at 20 °C. The Substrate Mixture (25 µL/well), containing the SARS-CoV-2 nsp2/3 cleavage site-derived peptide **2** (4.0 µM; VTNNTFTLKGG/APTKVTFGD, Supporting Figure S1), the *N*-acetylated N-terminal product peptide (0.4 µM; Ac-VTNNTFTLKGG, Supporting Figure S1), and the *N*-acetylated C-terminal product peptide (0.4 µM; Ac-APTKVTFGD, Supporting Figure S1) in 50 mM Tris buffer (pH 8.0), was added using the multidrop dispenser. The plates were centrifuged (1000 rpm, 5 s) and after incubating for 2 h at 37 °C, the enzyme reaction was stopped by addition of $10\%_{v/v}$ aqueous formic acid (5 µL/well). The plates were then centrifuged (1000 rpm, 10 s) and analyzed by MS.

MS-analyses were performed using a RapidFire RF 365 high-throughput sampling robot (Agilent) attached to an iFunnel Agilent 6550 accurate mass quadrupole time-of-flight (Q-TOF) mass spectrometer operated as described in the Supporting Information (Section 4). For data analysis, the m/z +1 charge states of both the C-terminal and the N-terminal product peptides, as well as the corresponding *N*-acetylated C-terminal and N-terminal product peptides, which were used as internal standards, were used to extract ion chromatogram data; peak areas were integrated using the RapidFire Integrator software (Agilent). Data were exported into Microsoft Excel and used to calculate the averaged product peptide concentrations using the equation: averaged product peptide concentrations = (0.2 µM × (integral C-terminal product peptide) / (integral *N*-acetylated C-terminal product peptide) + 0.2 µM × (integral N-terminal product peptide) / (integral *N*-acetylated N-terminal product peptide)) / 2. Normalized dose-response curves (DMSO and formic acid controls) were obtained from the raw data by non-linear regression (GraphPad Prism 5) and used to determine IC_{50}-values. The standard deviation (SD) of two independent IC_{50} determinations (n = 2) was calculated using GraphPad Prism 5. Z′-factors were calculated according to the literature using Microsoft Excel.[4]

6. References

[1] National Center for Biotechnology Information (NCBI) reference sequence: NC_045512.2; https://www.ncbi.nlm.nih.gov/nuccore/NC_045512

[2] a) C. T. Lim, K. W. Tan, M. Wu, R. Ulferts, L. A. Armstrong, E. Ozono, L. S. Drury, J. C. Milligan, T. U. Zeisner, J. Zeng, F. Weissmann, B. Canal, G. Bineva-Todd, M. Howell, N. O'Reilly, R. Beale, Y. Kulathu, K. Labib, J. F. X. Diffley, *Biochem. J.* **2021**, *478*, 2517-2531; b) L. A. Armstrong, S. M. Lange, V. Dee Cesare, S. P. Matthews, R. S. Nirujogi, I. Cole, A. Hope, F. Cunningham, R. Toth, R. Mukherjee, D. Bojkova, F. Gruber, D. Gray, P. G. Wyatt, J. Cinatl, I. Dikic, P. Davies, Y. Kulathu, *PLoS One* **2021**, *16*, e0253364; c) C.-J. Kuo, T.-L. Chao, H.-C. Kao, Y.-M. Tsai, Y.-K. Liu, L. H.-C. Wang, M.-C. Hsieh, S.-Y. Chang, P.-H. Liang, *Antimicrob. Agents Chemother.* **2021**, *65*, e02577-20.

[3] D. Shin, R. Mukherjee, D. Grewe, D. Bojkova, K. Baek, A. Bhattacharya, L. Schulz, M. Widera, A. R. Mehdipour, G. Tascher, P. P. Geurink, A. Wilhelm, G. J. van der Heden van Noort, H. Ovaa, S. Müller, K.-P. Knobeloch, K. Rajalingam, B. A. Schulman, J. Cinatl, G. Hummer, S. Ciesek, I. Dikic, *Nature* **2020**, *587*, 657-662.

[4] J.-H. Zhang, T. D. Y. Chung, K. R. Oldenburg, *J. Biomol. Screen.* **1999**, *4*, 67-73.
